# A Shell Model for Free Vibration Analysis of Carbon Nanoscroll

**DOI:** 10.3390/ma10040387

**Published:** 2017-04-06

**Authors:** Amin Taraghi Osguei, Mohamad Taghi Ahmadian, Mohsen Asghari, Nicola Maria Pugno

**Affiliations:** 1 Department of Mechanical Engineering, Sharif University of Technology, 11365-11155 Tehran, Iran; taraghi@mech.sharif.ir (A.T.O.); asghari@sharif.edu (M.A.); 2 Center of Excellence in Design, Robotics and Automation (CEDRA), Sharif University of Technology, 11365-9567 Tehran, Iran; 3 Laboratory of Bio-Inspired and Graphene Nanomechanics, Department of Civil, Environmental and Mechanical Engineering, University of Trento, 38123 Trento, Italy; nicola.pugno@unitn.it; 4 School of Engineering and Materials Science, Queen Mary University of London, Mile End Road, London E1 4NS, UK; 5 Ket-Lab, Italian Space Agency, Via del Politecnico s.n.c., 00133 Rome, Italy

**Keywords:** carbon nanoscroll, shell modeling, natural frequency, arbitrary boundary condition, van der Walls interactions

## Abstract

Carbon nanoscroll (CNS) is a graphene sheet rolled into a spiral structure with great potential for different applications in nanotechnology. In this paper, an equivalent open shell model is presented to study the vibration behavior of a CNS with arbitrary boundary conditions. The equivalent parameters used for modeling the carbon nanotubes are implemented to simulate the CNS. The interactions between the layers of CNS due to van der Waals forces are included in the model. The uniformly distributed translational and torsional springs along the boundaries are considered to achieve a unified solution for different boundary conditions. To study the vibration characteristics of CNS, total energy including strain energy, kinetic energy, and van der Waals energy are minimized using the Rayleigh-Ritz technique. The first-order shear deformation theory has been utilized to model the shell. Chebyshev polynomials of first kind are used to obtain the eigenvalue matrices. The natural frequencies and corresponding mode shapes of CNS in different boundary conditions are evaluated. The effect of electric field in axial direction on the natural frequencies and mode shapes of CNS is investigated. The results indicate that, as the electric field increases, the natural frequencies decrease.

## 1. Introduction

Rolling a monolayer graphene sheet into a spiral multilayer structure forms a roll-like nanostructure called CNS. The open configuration of CNS contains two boundaries along the curved edges and two boundaries along axial direction. Bacon introduced CNSs as a kind of graphite whiskers for the first time in 1960 [1]. However, CNSs received high attention after discovering a new production procedure in 2003 [2]. In recent decade, numerous researchers have been trying to develop more accurate and controlled methods for synthesis of CNS [3,4,5,6]. CNSs are successfully used as biosensors [7], water and ion channels [8], supercapacitors [9], and hydrogen storage devices [10,11,12].

Although CNSs are very similar to multi-wall carbon nanotubes (MWCNT), their open topology indicates specific characteristics distinct from well-known carbon nanotubes (CNTs). The open structure of CNS makes it possible to design new instruments based on its tunable core by controlling relative sliding between layers. Shi et al. studied equilibrium core radius of CNS by balancing elastic bending energy and van der Waals interaction energy and investigated the constitutive behavior of CNS in response to inner and external pressure [13,14]. A comparative study on the electrical and mechanical behavior of MWCNT and CNS shows higher flexibility of scroll structure [15]. Zaeri and Ziaei-Rad indicated that the stiffness of the CNS under tension is similar to that of CNT. However, torsional stiffness of CNS is highly dependent on the van der Waals interactions [16]. Shi et al. developed a theoretical model to investigate the mechanical behavior of CNS crystals subjected to uniaxial lateral compression [17]. This research indicates promising application of CNSs in energy-absorbing materials such as artificial muscles. Zhang et al. studied buckling instability of CNSs under axial compression, torsion and bending using molecular mechanics simulations and clarified the difference in the critical buckling condition between CNSs and MWCNTs [18]. The translational rolling and unrolling of a CNS on a rigid substrate was investigated [19]. This study demonstrates the potential of CNSs for application as actuators.

Although the vibration analysis of CNT is widely studied and reported in the literature, there are only limited reports on the vibration of CNSs. Shi et al. investigated CNS as a gigahertz breathing oscillator [20]. Cheng et al. compared the oscillatory motion of MWCNT and CNS on a substrate using molecular dynamic (MD) simulations [21]. Results show that the motion of CNS-based oscillator is controllable and offers advantages over MWCNT-based oscillator on substrate.

Modal analysis of nanostructures is essential for device applications like sensors and actuators. In this regard, modal analysis of CNT, carbon nanocone, fullerene, graphene, and other carbon-based nanostructures are conducted using different methods. For instance, vibrational analysis of fullerene is studied using MD simulations [22] and finite element method (FEM) is used to perform modal analysis of CNT and carbon nanocone [23]. However, one of the most efficient methods for vibration analysis of nanostructures is the continuum-based technique.

Based on concepts of elasticity, the mechanical behavior of nanostructures, especially CNTs, is widely studied and reported in the literature [24]. For instance, researchers studied the buckling behavior and vibrational characteristics of CNTs using shell models [25,26,27,28]. These numerically efficient models help researchers for better understanding and interpretation of the experimental results. Peng et al. examined the possibility of modeling a CNT with thin shell and estimated the order of error in representing the single-wall carbon nanotube (SWCNT) by a continuum thin shell [29]. Wang et al. investigated the applicability and limitation of simplified elastic shell equations for CNTs [30]. Generally, for buckling and vibration analysis of CNTs, the reports show that simplified Flugge model is more accurate than Donnell’s model. Silvestre studied the accuracy and suitability of shell models for torsional buckling of CNTs [31]. The findings of this research indicate Sanders shell model leads to correct results, while Donnell shell theory fails to predict correct behavior.

Beside various studies on different shell theories and their applicability and accuracy, some researchers developed equivalent continuum models considering hexagonal lattice structure of CNTs. Ru proposed the effective bending stiffness of a SWCNT [32]. Vodenitcharova predicted effective wall thickness of a CNT using the ring theory of continuum mechanics [33]. Odegard et al. proposed an equivalent continuum model for nanostructures to serve a link between computational chemistry and solid mechanics [34]. Zhang established a nanoscale continuum for predicting the Young’s Modulus of SWCNT based on the interatomic potential of Carbon atoms [35]. Chang presented a molecular-based anisotropic shell model of SWCNTs using a molecular mechanics [36]. Including the size effect in elastic continuum shell models for analyzing nanoscale structures is an important issue which should be considered [37]. The influence of size effect on the natural frequencies of a nanostructure appears in high frequencies and since this study is limited to low frequency vibrations, the size effects are neglected.

One of the challenges in modeling of MWCNTs arises when each of the nested tubes are considered as individual cylinders. Therefore, the van der Waals forces interacting between layers of MWCNT which come from interlayer displacements should be modeled. Ru presented an elastic model for column buckling of a MWCNT by considering intertube van der Waals forces [38]. Saito et al. studied the stable structure of double-wall carbon nanotube (DWCNT) [39]. Based on the data of reference [39], Sudak assumed the van der Waals pressure at any point between adjacent tubes should be a linear function of the jump in deflection at that point and used this model for linearized infinitesimal buckling analysis [40]. However, Zhang et al. implemented this model to evaluate the transverse vibration of MWCNTs [41].

There are numerous studies on the free vibration of SWCNT [42,43], DWCNT [44], and MWCNT [45] using continuum shell models during the last decade. In addition researchers investigated boundary effect [46,47], coupling between flexural modes [48], nonlinear vibration [49,50,51], wave propagation [52,53], and interlayer degree of freedom [54]. However, these models could not be applied for predicting vibrational behavior of CNS due to technical limitations. The morphology of CNSs makes it impossible to consider each cycle of CNS as a beam and model the van der Waals forces between layers.

In the present study, a model for free vibration analysis of CNS with different boundary conditions is proposed. The model enables researchers to understand the vibrational behavior of CNS at different boundary conditions and study the effect of each parameter on natural frequencies. After considering CNS as an equivalent shell, uniformly distributed springs along each boundary are added to simulate the resultant forces in that boundary condition. The equations of motion are derived based on First Order Shear Deformation Theory (FSDT) by considering Kirchhoff-Love assumptions. The solution is obtained using Rayleigh-Ritz technique by selecting Chebyshev orthogonal polynomial of first kind as admissible displacement functions. As an example, the natural frequencies and mode shapes of a few CNSs with different boundary conditions are reported in this study. Additionally, the effect of van der Waals forces on the vibration characteristics of CNS is investigated.

## 2. Modeling

This work deals with modeling vibration behavior of a CNS subjected to arbitrary boundary condition using equivalent continuum thin shell theory. Based on the thin shell theories, the equivalent geometrical and mechanical properties of the CNS are required. The second step is determining equations of motion and solving them. Here, the energy expressions of the equivalent shell are obtained using FSDT and vibration characteristics are extracted through Rayleigh-Ritz procedure. In order to use a unified formulation for vibration behavior of CNSs in arbitrary boundary conditions, the energy stored in boundary conditions is modeled with uniformly distributed springs along each boundary.

### 2.1. Equivalent Parameters

Different equivalent shell models are proposed for analyzing mechanical behavior of carbon-based nanostructures. For instance, in some studies, the distance between layers of graphite is assumed as wall thickness of equivalent shell and isotropic mechanical parameters are used. However, these equivalent models fail to accurately predict the bending rigidity of CNTs [42]. Previous studies show that the equivalent parameters E=5.5 TPa, ν=0.19, $\rho =11700\;kg/m^{3}$, and h=0.066 nm are suitable for modeling a SWCNT with equivalent continuum shell [42]. This mass density for CNT is calculated using equation, ρ=σ/h, which $\sigma =7.718\times 10^{-7}\;kg/m^{2}$ denotes the surface density of graphite. Accordingly, it is compatible with $\rho =2267\;kg/m^{3}$ reported for shell models with equivalent wall thickness of h=0.34 nm [20]. Therefore, CNSs which are constructed from rolling a graphene sheet may be modeled as an isotropic shell, like CNTs, by defining four parameters; i.e., Young modulus *E*, Poisson ratio ν, mass density ρ, and equivalent wall thickness of shell h.

**Figure 1:**
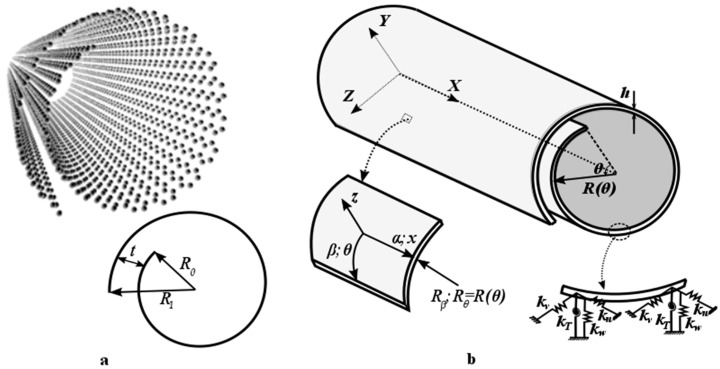
Geometry of a CNS: (**a**) atomistic model of CNS and its cross-section; (**b**) equivalent continuum shell model.

Figure 1 shows a CNS with length L and subtended angle ϕ, which can be subjected to an arbitrary boundary condition. The inner radius $R_{0}$, outer radius $R_{1}$, and interlayer distance between neighboring layers of CNS t, defines the configuration of cross section. The position of a point on the middle surface of equivalent shell model can be represented in the global Cartesian coordinate system (*XYZ*). However, the cylindrical coordinate system (α:x, β:θ, *z*) might be used for determining the energy expressions of the shell. u, v and w represent the displacement components in the directions of this coordinate system, respectively. $R_{\beta }:R_{\theta }$ shows the radius of curvature of the shell in circumferential direction of mid surface. Uniformly distributed translational springs ($k_{u}$, $k_{v}$, $k_{w}$) and the torsional spring ($k_{T}$) along the boundaries are considered to obtain a unified solution at different boundary conditions. A typical point in middle surface of equivalent thin shell can be defined as

(1) $$\vec{r}(x,\theta )=x\vec{I}+(R_{0}+a\theta )\cos\theta \;\vec{J}+(R_{0}+a\theta )\sin\theta \;\vec{K}$$

where $\vec{I}$, $\vec{J}$ and $\vec{K}$ denote the unit vectors along X, Y and Z axes. The parameter a which controls the distance between layers of CNS equals t/2π. By considering parameter a to be small, the Lame parameters which denote the length of the vectors $\partial \;\vec{r}/\partial \;x$ and $\partial \;\vec{r}/\partial \;\theta$ for the cylindrical panel with spiral cross section can be described as

(2.1) A=1

(2.2) $$B=\sqrt{a^{2}+{\left(R_{0}+a\theta \right)}^{2}}\approx R_{0}+a\theta$$

The curvature radius of the shell along *x* direction equals infinity and along tangential direction could be determined by

(3) $$R_{\theta }=\frac{\sqrt{{\left(a^{2}+{\left(R_{0}+a\theta \right)}^{2}\right)}^{3}}}{2a^{2}+{\left(R_{0}+a\theta \right)}^{2}}\approx R_{0}+a\theta$$

### 2.2. Continuum Shell Theory

This study investigates low frequency vibration of CNS with large aspect ratio. The thickness of equivalent shell is small and any elastic shell theory could be applied to analyze the vibration of CNS. However, in this study, FSDT based on Love’s approximations is used for modeling CNS. Strain at a point on the equivalent shell using FSDT by considering membrane and bending strains could be expressed as

(4) $$\begin{array}{l} \varepsilon_{xx}=\varepsilon_{xx}^{0}+z\;k_{xx}\\ \varepsilon_{\theta \theta }=\varepsilon_{\theta \theta }^{0}+z\;k_{\theta \theta }\\ \gamma_{x\theta }=\gamma_{x\theta }^{0}+z\;k_{x\theta } \end{array}$$

in which $\varepsilon_{xx}^{0}$, $\varepsilon_{\theta \theta }^{0}$ and $\gamma_{x\theta }^{0}$ are normal and shear strains at middle surface of the CNS. $k_{xx}$, $k_{\theta \theta }$ and $k_{x\theta }$ denote curvature changes and twist at middle surface, respectively. Considering the geometric parameters of CNS, the strain-displacement equations of the middle surface are

(5) $$\begin{array}{l} \varepsilon_{xx}^{0}=\frac{\partial u}{\partial x};\;\varepsilon_{\theta \theta }^{0}=\frac{1}{R_{0}+a\theta }\frac{\partial v}{\partial \beta }+\frac{w}{R_{\theta }};\;\gamma_{x\theta }^{0}=\frac{\partial v}{\partial x}+\frac{1}{R_{0}+a\theta }\frac{\partial u}{\partial \theta }\\ k_{xx}=-\frac{\partial^{2}w}{\partial x^{2}};\;k_{\theta \theta }=\frac{1}{{\left(R_{0}+a\theta \right)}^{2}}\frac{\partial v}{\partial \theta }-\frac{1}{{\left(R_{0}+a\theta \right)}^{3}}\frac{\partial R_{\theta }}{\partial \theta }v+\frac{1}{{\left(R_{0}+a\theta \right)}^{3}}\frac{\partial B}{\partial \theta }\frac{\partial w}{\partial \theta }-\frac{1}{{\left(R_{0}+a\theta \right)}^{2}}\frac{\partial^{2}w}{\partial \theta^{2}}\\ k_{x\theta }=\frac{1}{R_{0}+a\theta }\frac{\partial v}{\partial x}-\frac{2}{R_{0}+a\theta }\frac{\partial^{2}w}{\partial x\;\partial \theta } \end{array}$$

Generalized Hook’s law describes the equation between components of stress and strain at a typical point. Force and moment applied on the equivalent shell model of a CNS could be calculated by integrating components of stress along the thickness of a shell as

(6) $$N_{xx}=\frac{Eh}{1-\nu^{2}}\varepsilon_{xx}^{0}+\frac{\nu Eh}{1-\nu^{2}}\varepsilon_{\theta \theta }^{0};\;N_{\theta \theta }=\frac{\nu Eh}{1-\nu^{2}}\varepsilon_{xx}^{0}+\frac{Eh}{1-\nu^{2}}\varepsilon_{\theta \theta }^{0};\;N_{x\theta }=\frac{Eh}{2(1+\nu )}\gamma_{x\theta }$$

(7) $$M_{xx}=\frac{Eh^{3}}{12(1-\nu^{2})}k_{xx}+\frac{\nu Eh^{3}}{12(1-\nu^{2})}k_{\theta \theta };\;M_{\theta \theta }=\frac{\nu Eh^{3}}{12(1-\nu^{2})}k_{xx}+\frac{Eh^{3}}{12(1-\nu^{2})}k_{\theta \theta };\;M_{x\theta }=\frac{Eh^{3}}{24(1+\nu )}k_{x\theta }$$

The total strain energy stored in the equivalent continuum shell of CNS could be calculated using Equations (5)–(7).

(8) $$U_{shell}=\iint_{S}(N_{\;xx}\varepsilon_{xx}^{0}+N_{\;\theta \theta }\varepsilon_{\theta \theta }^{0}+N_{\;x\theta }\varepsilon_{x\theta }^{0}+M_{\;xx}k_{xx}+M_{\;\theta \theta }k_{\theta \theta }+M_{\;x\theta }k_{x\theta })ds$$

The corresponding kinetic energy of the shell can be written as

(9) $$T=\iint_{S}\frac{\rho h}{2}\left({\left(\frac{\partial u}{\partial \;t}\right)}^{2}+{\left(\frac{\partial v}{\partial \;t}\right)}^{2}+{\left(\frac{\partial w}{\partial \;t}\right)}^{2}\right)ds$$

where ρ denotes the mass density of the shell.

### 2.3. Van der Waals Interactions

The most applicable models of van der Waals forces are developed based on Lennard Jones potential considering inter atomic interactions. For linearized column buckling and small-deflection linear vibration of MWCNT, the pressure at any point between two adjacent layers of MWCNTs is a linear function of the jump in deflection at that point [38,40,41].

(10) $$P_{ij}=c(w_{i}-w_{j})$$

where *c* is the intertube interaction coefficient per unit length between two layers of MWCNT and can be defined as [41]

(11) $$c=\frac{320\times 2R_{in}}{0.16d^{2}}\;(erg/cm^{3})\;,\;d=1.42\times 10^{-8}\;cm$$

The coefficient easily can be calculated for a specified inner radius $R_{in}$, and lattice constant *d*. The essential requirement for modeling van der Waals interactions of CNS is defining pressure per unit area at any point. Thus, one can obtain the interlayer interaction coefficient per unit area by $D_{vdw}=c/(2\pi R_{in})$. The total energy expression for van der Waals interaction between successive layers of CNS is the integration of energy at a point due to deflection of successive layers.

(12) $$U_{vdw}=\frac{1}{2}\int_{0}^{L}\int_{0}^{\phi -2\pi }D_{vdw}{\left(w(x,\theta +2\pi )-w(x,\theta )\right)}^{2}(R_{0}+a\theta )d\theta dx$$

where w(x,θ) represents the deflection of a typical point at arbitrary layer. Equation (12) implies that the first and last layers interact only with one layer, while middle layers interact with two layers.

### 2.4. Boundary Conditions

Equivalent shell model of CNS, unlike MWCNT, is an open shell which consists of four boundary conditions. The open topology of CNS creates more difficulties comparing with CNT in vibration analysis. A set of uniformly distributed translational and torsional springs are considered along each boundary to study the vibration characteristic of CNS. These springs enable us to achieve a unified solution for different boundary conditions and study non-classical boundaries. The energy stored in the boundary springs which simulates the boundary conditions effect is

(13) $$\begin{array}{cc} U_{b} & =\int_{0}^{\phi }\left\{{\left[k_{u}^{x_{0}}u^{2}+k_{v}^{x_{0}}v^{2}+k_{w}^{x_{0}}w^{2}+k_{T}^{x_{0}}{\left(\frac{\partial w}{\partial x}\right)}^{2}\right]}_{x=0}+{\left[k_{u}^{x_{L}}u^{2}+k_{v}^{x_{L}}v^{2}+k_{w}^{x_{L}}w^{2}+k_{T}^{x_{L}}{\left(\frac{\partial w}{\partial x}\right)}^{2}\right]}_{x=L}\right\}(R_{0}+a\theta )d\theta \\ & +\int_{0}^{L}\left\{{\left[k_{u}^{\theta_{0}}u^{2}+k_{v}^{\theta_{0}}v^{2}+k_{w}^{\theta_{0}}w^{2}+k_{T}^{\theta_{0}}{\left(\frac{\partial w}{\partial \theta }\right)}^{2}\right]}_{\theta =0}+{\left[k_{u}^{\theta_{\phi }}u^{2}+k_{v}^{\theta_{\phi }}v^{2}+k_{w}^{\theta_{\phi }}w^{2}+k_{T}^{\theta_{\phi }}{\left(\frac{\partial w}{\partial \theta }\right)}^{2}\right]}_{\theta =\phi }\right\}dx \end{array}$$

in which $k_{i}^{j_{s}}\;i=u,v,w,T;\;j_{s}=x_{0},x_{L},\theta_{0},\theta_{\phi }$ represent the stiffness of springs located at boundary edge $j_{s}$. For example, $k_{w}^{x_{0}}$ shows the stiffness of spring in *z* direction located at boundary *x* = 0. The stiffness of four groups of springs should be determined based on boundary condition. Free boundary condition on the curved edge x=0 can be described as: $k_{u}^{x_{0}}=0,k_{v}^{x_{0}}=0,k_{w}^{x_{0}}=0,k_{T}^{x_{0}}=0$. Assuming $D=Eh^{3}/12(1-\upsilon^{2})$ as bending stiffness of the shell, other classical boundary conditions on the mentioned boundary can be represented as: simply support; $k_{u}^{x_{0}}=10^{9}D$, $k_{v}^{x_{0}}=10^{9}D$, $k_{w}^{x_{0}}=10^{9}D$, $k_{T}^{x_{0}}=0$, clamped; $k_{u}^{x_{0}}=10^{9}D$, $k_{v}^{x_{0}}=10^{9}D$, $k_{w}^{x_{0}}=10^{9}D$, $k_{T}^{x_{0}}=10^{9}D$, shear diaphragm; $k_{u}^{x_{0}}=0$, $k_{v}^{x_{0}}=10^{9}D$, $k_{w}^{x_{0}}=10^{9}D$, $k_{T}^{x_{0}}=0$. The values assigned for the stiffness of springs at boundaries are determined by analyzing the influence of boundary conditions on the shell vibration. Enhancement of spring stiffness beyond specified value is worthless and does not change the results. For defining the boundary condition at each edge, a simple letter string like FSCD is used. FSCD represents a CNS with free (F), simply support (S), clamped (C), and shear diaphragm (D) boundary conditions at the edges x=0, x=L, θ=0, and θ=ϕ, respectively.

### 2.5. Solution Method

The total Lagrangian energy for equivalent shell model of CNS can be determined using four energy expressions calculated in Equations (8), (9), (12) and (13) as follows

(14) $$L=T-U_{shell}-U_{vdw}-U_{b}$$

For extracting the natural frequencies and mode shapes of CNS, the Rayleigh-Ritz procedure is used. In the first step, the admissible functions of displacement components should be defined. Usually, boundary conditions limit the selection of appropriate admissible functions. However, adding the effects of boundary conditions in energy expressions makes it possible to select any independent and complete bases function. Here, the Chebyshev polynomials of the first kind are considered as admissible functions.

(15) $$\begin{array}{l} u(x,\theta ,t)=\sum_{m=0}^{\infty }\sum_{n=0}^{\infty }U_{mn}P_{m}(x)P_{n}(\theta )e^{j\;\omega t}\\ v(x,\theta ,t)=\sum_{m=0}^{\infty }\sum_{n=0}^{\infty }V_{mn}P_{m}(x)P_{n}(\theta )e^{j\;\omega t}\\ w(x,\theta ,t)=\sum_{m=0}^{\infty }\sum_{n=0}^{\infty }W_{mn}P_{m}(x)P_{n}(\theta )e^{j\;\omega t} \end{array}$$

where $U_{mn}$, $V_{mn}$ and $W_{mn}$ are unknown coefficients of displacement functions. $P_{m}(x)$ and $P_{n}(\theta )$ represent Chebyshev polynomials of first kind as follows

(16) $$\begin{array}{l} P_{0}(s)=1;\;P_{1}(s)=s\\ P_{i}(s)=2sP_{i-1}(s)-P_{i-2}(s)\;i>1 \end{array}$$

Chebyshev polynomials of first kind are defined on the interval [−1, 1]. In order to implement this procedure, dimensions of CNS need to be transformed. The coordinate transformation between dimensions of CNS and mentioned the interval is $\bar{x}=2x/L-1$, $\bar{\theta }=2\theta /\phi -1$.

Substituting admissible functions of CNS into energy expressions and minimizing total Lagrangian energy with respect to unknown coefficients of displacement construct a set of eigenvalues

(17) $$\left(K-\omega^{2}M\right)\Psi =0$$

where K and M are the generalized stiffness and mass matrices, respectively. The elements of these matrices are given in the Appendix A. Meanwhile, Ψ represents the undetermined coefficients of displacement functions and its transpose vector is $\Psi^{T}=[U^{T}\;V^{T}\;W^{T}]$. The transpose vector $U^{T}=[U_{00},...,U_{0N},U_{10},...,U_{1N},...,U_{M0},...,U_{MN}]$ represents the undefined coefficients of displacement function in *x* direction. The subscribes M and  N indicate the truncation point of admissible functions in x and θ directions, respectively. Vectors *V* and *W* can be specified similarly. In order to obtain natural frequencies of CNS, the eigenvalue problem presented in Equation (17) should be solved. The corresponding eigenvector of each natural frequency specifies mode shapes of CNS.

## 3. Results and Discussion

The geometrical structure of a CNS can be described by length, inner radius, separation distance, and subtended angle. However, instead of subtended angle, outer radius of cross section can be used for specifying the geometry of a CNS. The inner radius and subtended angle of a CNS in equilibrium condition can be extracted based on the equations of reference [13]. Distance between layers of CNSs is similar to that of graphite sheets and equals to 0.34 nm. Therefore, the cross section of a CNS is an Archimedean spiral which can be specified by inner radius and subtended angle. For example, based on the equations of reference [13], a typical CNS can be defined as: $R_{0}=0.6$ nm, $\phi =645^{\circ }$, and L=5 nm. In the following sections, vibration of this CNS would be investigated in different boundary conditions and for various lengths of CNS. Additionally, effect of van der Waals interactions on the vibration characteristics of CNS is studied.

### 3.1. Verification of the Model

#### 3.1.1. Convergence Study and Comparison between CNS and MWCNT

**Figure 2:**
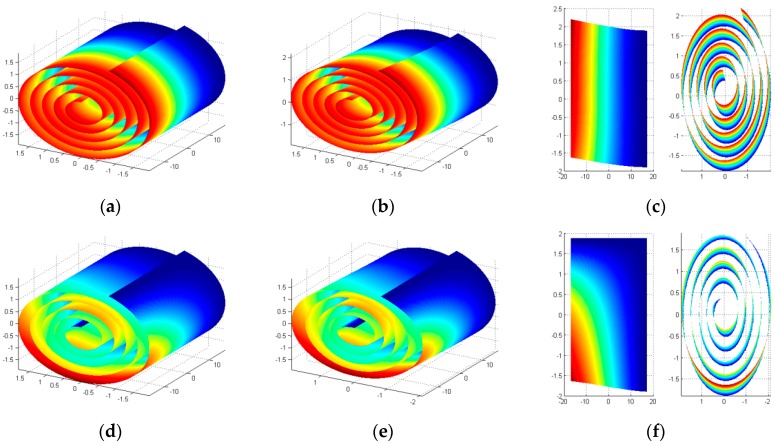
First mode shape of the CNS with CFFF boundary condition: (**a**) initial structure; (**b**) deformed structure; (**c**) front and side view. First mode shape of the CNS with CFCC boundary condition: (**d**) initial structure; (**e**) deformed structure; (**f**) front and side view.

Finding suitable results in the literature for the vibration behavior of CNS is a serious problem. Therefore, comparing the vibration characteristics of CNS with those of MWCNT is a possible solution. To carry out this investigation a stable CNS, $R_{0}=0.36$ nm, $\phi =1777^{\circ }$, L=34 nm, and an equivalent five-wall CNT with inner radius of 0.34 nm and length of 34 nm is considered. The first natural frequency of this MWCNT with all tubes clamped at one end is reported 68 GHz in the reference [45]. Unlike MWCNT, the CNS contains two extra boundaries parallel to the central axes. The first natural frequency of CNS for clamped (CFCC) and free (CFFF) boundary condition in these two boundaries is 10 GHz and 77 GHz, respectively. The corresponding mode shapes of these natural frequencies are depicted in Figure 2. However, the rigidity of MWCNT with one end clamped is between the rigidity of CNS with FCCC and FCFF boundaries. Therefore, the first natural frequency of MWCNT is between the first natural frequencies of CNS with FCCC and FCFF boundaries and the mode shape of CNS is similar to that of MWCNT reported in the reference [45]. Convergence of first 10 mode shapes of the CNS is reported in Table 1.

#### 3.1.2. The Breathing like Mode

**Figure 3:**
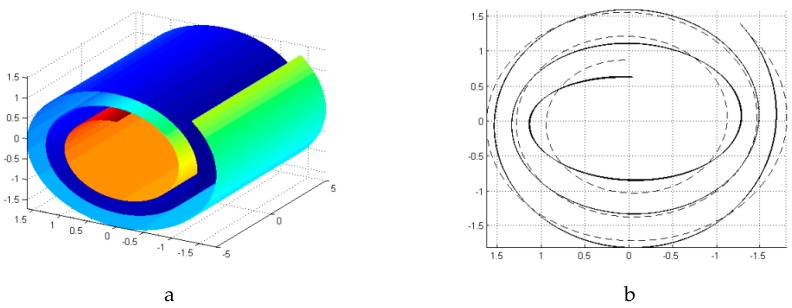
(**a**) Breathing like mode shape of the CNS; (**b**) the deformed cross section of CNS.

The breathing oscillatory motion studied in the literature is the only vibration behavior of the CNS which could be considered in this research. Shi et al. studied the breathing oscillation of the CNS by neglecting the radial displacement [20]. Their one degree freedom model estimates the oscillatory frequency of 20 GHz for a CNS defined as: $R_{0}=0.86$ nm, $\phi =1033^{\circ }$. In this research, the modal analysis of this CNS with different lengths is carried out. A breathing like mode shape of the CNS as shown in Figure 3 appears in the 8th, 11th and 25th mode of the CNS with length of 10 nm, 100 nm, and 500 nm, respectively. Interestingly, the natural frequencies of these mode shapes are same as each other and equal to 10 GHz which is comparable with oscillatory motion. Since, in this research, the CNS is not imposed to have only tangential displacement like oscillatory motion, the natural frequency is less than the oscillatory frequency.

### 3.2. Vibration of a CNS with Different Boundary Conditions

**Figure 4:**
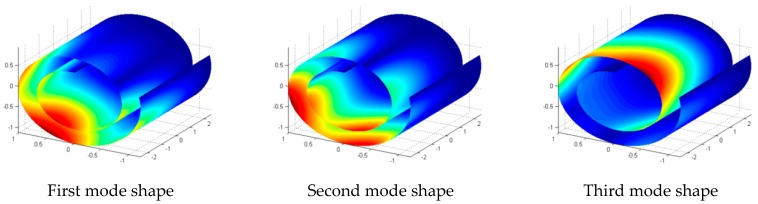

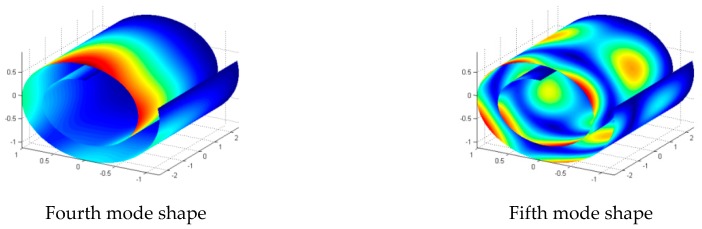
First five mode shapes of the CNS with FCSS boundary condition.

**Figure 5:**
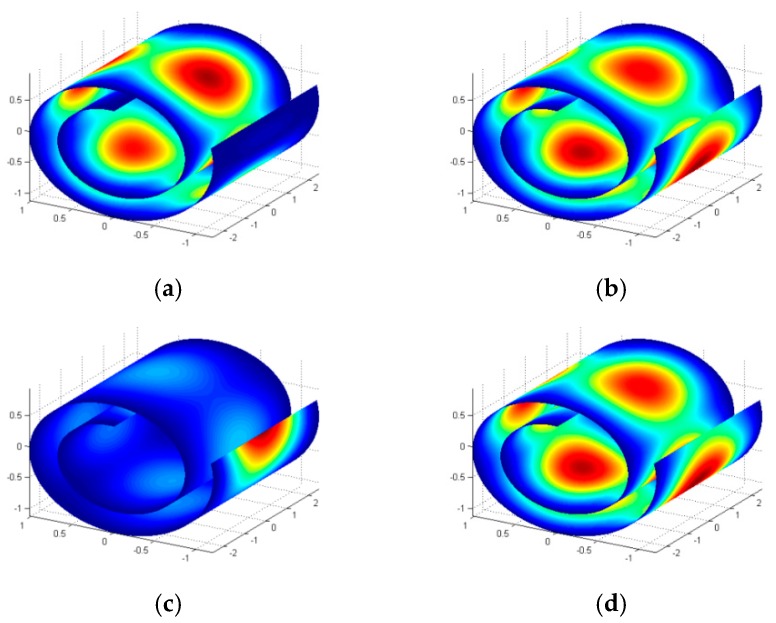
First mode shape of the CNS with different boundary condition: (**a**) CCCC; (**b**) SSDD; (**c**) CCFF; (**d**) DDDD.

Natural frequencies and corresponding mode shapes of the predefined CNS: $R_{0}=0.6$ nm, $\phi =645^{\circ }$, and L=5 nm at some classical boundary conditions are reported in this section. Table 2 represents first five natural frequencies of the CNS in various boundary conditions. As it was expected, the highest natural frequencies are obtained for CCCC boundary condition since a CNS with this boundary condition is more constrained than other boundary conditions. However, investigations show that boundary conditions at curved edges represent a more considerable effect on natural frequencies. Figure 4 represents first five mode shapes of the CNS with FCSS boundary condition and Figure 5 displays first mode shape of the CNS in other boundary conditions listed in Table 2.

### 3.3. Vibration of a CNS with Different Lengths

**Figure 6:**
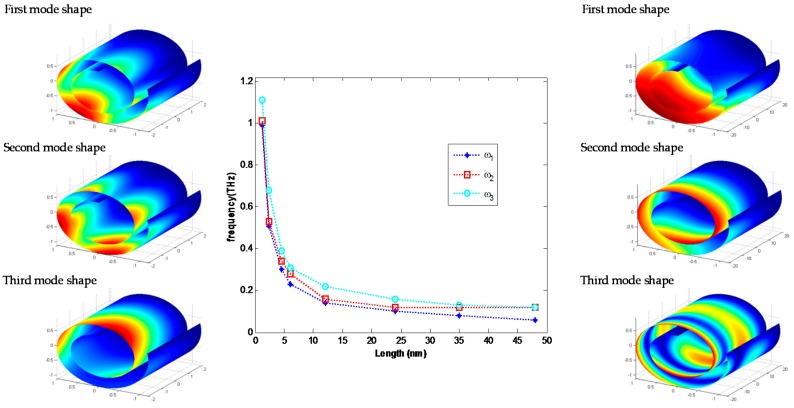
Variation of first three natural frequencies versus length of CNS with FCSS boundary condition.

Length of a cylinder is one of the most effective parameters in variation of natural frequencies due to change of mode shapes. Increasing the length of CNS decreases the value of natural frequencies and changes the mode shapes by decreasing number of wave along circumferential direction and increasing number of waves along axial direction. For a specific mode shape of CNSs as open noncircular cylinders, the number of waves in two directions depends on the boundary conditions. However, in general, increasing the length of a CNS leads to reduction in natural frequencies and change in mode shapes. For instance, Figure 6 shows the variation of first three natural frequencies in FCSS boundary condition as an example. In first three modes of a CNS with length 4 nm, the number of waves in circumfrential direction changes. In contrast, for mode shapes of a CNS with length 40 nm, the number of waves in the axial direction differs.

### 3.4. Effect of Van der Waals Interactions on the Vibration of a CNS

The energy derived from van der Waals (vdW) interactions between layers of a CNS can be controlled by applying electric field [8]. The applied electric field causes the carbon atoms to be polarized and decreases the effective energy between layers. Therefore, variation of electric field in the admissible range changes van der Waals energy considerably and alters the core size of CNS consequently.

**Figure 7:**
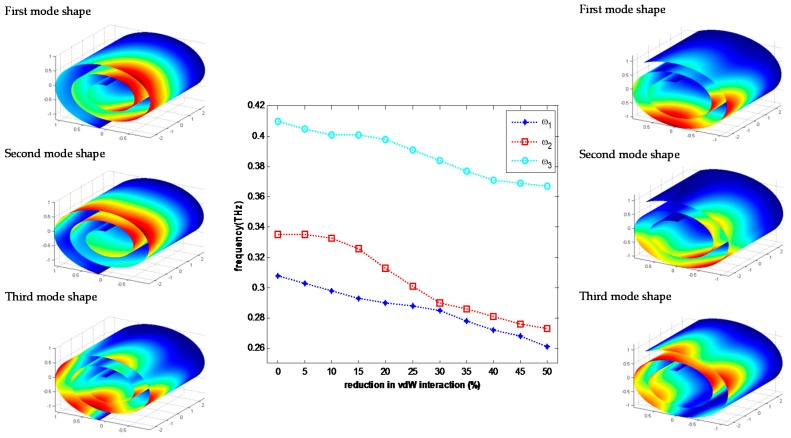
Variation of first three natural frequencies versus reduction in vdW interaction with FCSS boundary condition.

In order to study the effect of vdW energy on the natural frequencies of a CNS, a graphene sheet, 5 nm × 11.8 nm, has been considered. Rolling up this graphene sheet construct a CNS which its geometric parameters are: $R_{0}=0.34$ nm, $\phi =893^{\circ }$, and L=5 nm. Applying a DC electric field along the axial direction of CNS reduces the interlayer surface energy and increases the core size of CNS. For instance, diminishing 50% of the vdW energy using electric field results to a CNS as: $R_{0}=0.54$ nm, $\phi =755^{\circ }$, and L=5 nm. Figure 7 represents first three natural frequencies and mode shapes of CNS for these two configurations. Additionally, this figure displays the first three natural frequencies of CNS for every 5% reduction of vdW energy. To this end, in each step the equilibrium configuration of CNS is extracted and the natural frequencies of this structure are calculated. More reduction in vdW energy is meaningless since it can lead to the collapse of CNS.

## 4. Conclusions

A shell model based on FSDT is developed to study the vibration of CNSs. Rayleigh-Ritz method is used to determine the vibration characteristics. The unified model presented in this study enables the researchers to investigate the effect of boundary conditions on the vibration of a CNS.

The results show that restricting a CNS at two curved edges leads to local deflections along the straight boundaries. However, generally for other boundary conditions waves along the circumferential direction of CNSs can be observed. The vibration of a CNS cantilever as a target drug delivery system is studied thoroughly. Increasing the length of CNS cantilever decreases the natural frequencies and the number of waves in circumferential direction reduces. It should also be noted that as the length of CNS increases, the number of waves along the axial direction also increases. Applying electric field along the axial direction of CNS results in the enhancement of inner reduce and reduction of natural frequency. 

## Figures and Tables

**Table 1:** Convergence of the first 10 natural frequencies (G Hz) of the CNS.

Boundary Condition		CFFF			CFCC				
M × N		20 × 20	25 × 25	27 × 27	20 × 20	22 × 22	25 × 25	27 × 27	28 × 28
**Mode Shape**	1	17	10	10	83	79	77	77	77
	2	27	11	11	112	112	111	111	111
	3	48	48	48	130	127	123	121	121
	4	60	58	58	138	134	127	126	126
	5	66	62	62	163	160	150	147	147
	6	70	70	70	201	200	197	196	196
	7	147	146	146	213	211	208	207	207
	8	147	147	147	225	223	217	215	215
	9	151	151	151	244	243	241	240	240
	10	156	154	154	275	275	274	274	274

**Table 2:** First five natural frequencies (T Hz) of the CNS with different boundary conditions.

Mode Shape	Boundary Condition				
	CCCC	SSDD	CCFF	DDDD	FCSS
1	0.59	0.52	0.44	0.36	0.27
2	0.60	0.56	0.49	0.43	0.33
3	0.72	0.68	0.62	0.56	0.36
4	0.73	0.71	0.65	0.63	0.40
5	0.88	0.80	0.65	0.65	0.60

## Appendix A

The generalized stiffness and mass matrices are constructed from symmetric sub-matrices as follows

$$\begin{array}{l} K=\left[K_{uu}\;K_{uv}\;K_{uw}\;;\;K_{vu}\;K_{vv}\;K_{vw}\;;\;K_{wu}\;K_{wv}\;K_{ww}\;\right]\\ M=\left[M_{uu}\;0\;0\;;\;0\;M_{vv}\;0\;;\;0\;0\;M_{ww}\;\right] \end{array}$$

where $K_{ij},\;M_{ij};\;i,j=u,v,w$ denote sub-matrices. Elements of the sub-matrices can be determined using following expressions

$$\begin{array}{ll} {\left(k_{uu}\right)}_{ij}= & A_{11}II^{11}I_{1}^{00}+A_{66}II^{00}I_{-1}^{11}+k_{u}^{x_{0}}{\left(-1\right)}^{m+p}I_{1}^{00}+k_{u}^{x_{L}}I_{1}^{00}+k_{u}^{\theta_{0}}{\left(-1\right)}^{n+q}II^{00}+k_{u}^{\theta_{\phi }}II^{00}\\ {\left(k_{uv}\right)}_{ij}= & A_{12}II^{10}I_{0}^{01}+A_{66}II^{01}I_{0}^{10}\\ {\left(k_{uw}\right)}_{ij}= & A_{12}II^{10}I_{0}^{00}\\ {\left(k_{vv}\right)}_{ij}= & A_{22}II^{00}I_{-1}^{11}+A_{66}II^{11}I_{1}^{00}+D_{22}II^{00}I_{-3}^{11}-aD_{22}II^{00}I_{-4}^{01}-aD_{22}II^{00}I_{-4}^{10}+a^{2}D_{22}II^{00}I_{-5}^{00}\\ & +D_{66}II^{11}I_{-1}^{00}+k_{v}^{x_{0}}{\left(-1\right)}^{m+p}I_{1}^{00}+k_{v}^{x_{L}}I_{1}^{00}+k_{v}^{\theta_{0}}{\left(-1\right)}^{n+q}II^{00}+k_{v}^{\theta_{\phi }}II^{00}\\ {\left(k_{vw}\right)}_{ij}= & A_{22}II^{00}I_{-1}^{10}-D_{12}II^{02}I_{-1}^{10}+aD_{12}II^{02}I_{-2}^{00}+aD_{22}II^{00}I_{-4}^{11}-D_{22}II^{00}I_{-3}^{12}-a^{2}D_{22}II^{00}I_{-5}^{01}\\ & +aD_{22}II^{00}I_{-4}^{02}-2D_{66}II^{11}I_{-1}^{01}\\ {\left(k_{ww}\right)}_{ij}= & A_{22}II^{00}I_{-1}^{00}+D_{11}II^{22}I_{1}^{00}-aD_{12}II^{20}I_{-2}^{01}+D_{12}II^{20}I_{-1}^{02}-aD_{12}II^{02}I_{-2}^{10}+D_{12}II^{02}I_{-1}^{20}\\ & +a^{2}D_{22}II^{00}I_{-5}^{11}-aD_{22}II^{00}I_{-4}^{12}-aD_{22}II^{00}I_{-4}^{21}+D_{22}II^{00}I_{-3}^{22}+4D_{66}II^{11}I_{-1}^{11}+D_{vdw}II_{2}^{00}\\ & -D_{vdw}II_{2}^{0^{+}0}-D_{vdw}II_{2}^{00^{+}}+D_{vdw}II_{2}^{0^{+}0^{+}}+k_{w}^{x_{0}}{\left(-1\right)}^{m+p}I_{1}^{00}+k_{w}^{x_{L}}I_{1}^{00}+k_{w}^{\theta_{0}}{\left(-1\right)}^{n+q}II^{00}\\ & +k_{w}^{\theta_{\phi }}II^{00}+\frac{4}{L^{2}}k_{T}^{x_{0}}m^{2}p^{2}{\left(-1\right)}^{m+p}I_{1}^{00}+\frac{4}{L^{2}}k_{T}^{x_{L}}m^{2}p^{2}I_{1}^{00}+\frac{4}{{\theta_{0}}^{2}}k_{T}^{\theta_{0}}n^{2}q^{2}{\left(-1\right)}^{n+q}II^{00}\\ & +\frac{4}{{\theta_{0}}^{2}}k_{T}^{\theta_{\phi }}n^{2}q^{2}II^{00}\\ {\left(M_{uu}\right)}_{ij}= & {\left(M_{vv}\right)}_{ij}={\left(M_{ww}\right)}_{ij}=\rho hII^{00}I_{1}^{00} \end{array}$$

where *i* and *j* are predefined indexes as: i=m(N+1)+n+1, j=p(N+1)+q+1. The coefficients used in determination of matrices for eigenvalue problem can be determined by

$$\begin{array}{l} II^{ab}=\int_{0}^{L}\frac{d^{a}P_{m}(x)}{dx^{a}}\frac{d^{b}P_{p}(x)}{dx^{b}}dx;\;I_{c}^{ab}=\int_{0}^{\phi }\frac{d^{a}P_{n}(\theta )}{d\theta^{a}}\frac{d^{b}P_{q}(\theta )}{d\theta^{b}}.{\left(R_{0}+a\theta \right)}^{c}d\theta \\ I_{c}^{a^{+}b}=\int_{0}^{\phi -2\pi }\frac{d^{a}P_{n}(\theta +2\pi )}{d\theta^{a}}\frac{d^{b}P_{q}(\theta )}{d\theta^{b}}.{\left(R_{0}+a\theta \right)}^{c}d\theta \\ A_{11}=A_{22}=\frac{Eh}{1-\upsilon^{2}};\;A_{12}=\frac{\upsilon Eh}{1-\upsilon^{2}};\;A_{66}=\frac{Eh}{2(1+\upsilon )}\\ D_{11}=D_{22}=\frac{Eh^{3}}{12(1-\upsilon^{2})};\;D_{12}=\frac{\upsilon Eh^{3}}{12(1-\upsilon^{2})};\;D_{66}=\frac{Eh^{3}}{24(1+\upsilon )} \end{array}$$
